# Effect on nutritional status and biomarkers of inflammation and oxidation of an oral nutritional supplement (with or without probiotics) in malnourished hemodialysis patients. A multicenter randomized clinical trial “Renacare Trial”

**DOI:** 10.3389/fnut.2023.1107869

**Published:** 2023-02-03

**Authors:** Francisco Hevilla, Marina Padial, María Blanca, Guillermina Barril, Tamara Jiménez-Salcedo, Mercedes Ramirez-Ortiz, Ángel Nogueira, Adriana Gentile, Eva García-Escobar, Silvana Y. Romero-Zerbo, Gabriel Olveira

**Affiliations:** ^1^Servicio de Endocrinología y Nutrición, Instituto de Investigación Biomédica de Málaga–Plataforma BIONAND, Hospital Regional Universitario de Málaga, Málaga, Spain; ^2^Departamento de Medicina y Dermatología, Universidad de Málaga, Málaga, Spain; ^3^Servicio de Endocrinología y Nutrición, Hospital Universitario Rey Juan Carlos, Madrid, Spain; ^4^Servicio de Nefrología, Hospital de la Princesa, Madrid, Spain; ^5^Servicio de Nefrología, Hospital Regional Universitario de Málaga, Málaga, Spain; ^6^CIBER de Diabetes y Enfermedades Metabólicas Asociadas, Instituto de Salud Carlos III, Málaga, Spain

**Keywords:** oral nutritional supplement, hemodialysis, inflammation biomarkers, oxidation biomarkers, malnutrition, probiotics

## Abstract

**Background:**

Malnutrition in patients undergoing hemodialysis is frequent and associated with a reduction in muscular mass and strength, with an increment in biomarkers of inflammation and oxidation.

**Materials and methods:**

Randomized, multicenter, parallel-group trial in malnourished hemodialysis patients with three groups [(1) control (C) individualized diet, (2) oral nutritional supplement-ONS- + placebo-SU- PL-, and (3) ONS + probiotics-SU-PR]; the trial was open regarding the intake of ONS or individualized diet recommendations, but double-blind for the intake of probiotics. We obtained, at baseline and after 3 and 6 months, anthropometric measurements, handgrip strength, bioelectrical impedance analysis (BIA), dietary records, and routine biochemical parameters. Inflammation and oxidation were determined using ELISA techniques (Versamax and ProcartaPlex multiplex Immunoassay). Results were analyzed by intention to treat.

**Results:**

A total of 31 patients (11 corresponding to group C, 10 to SU-PL, and 10 to SU-PR) completed the 6-months trial. The two groups that took supplements significantly increased their protein calorie, fat (total and n-3), and fiber intake. Weight and fat-free mass (FFM) also increased significantly in the groups on supplements, both at 3 and 6 months, and dynamometry did so in the SU-PL group. At month 3, prealbumin and vitamin D were significantly increased in the SU-TOT (SU-PL + SU-PR) group. No changes were observed regarding levels of phosphorus and potassium in any of the groups. Urea increased significantly at 6 months in the SU-PL group. There were significant changes in some inflammation biomarkers in the groups on supplements during the intervention (brain-derived neurotrophic factor, bone morphogenetic protein-2, MCP-1, IL-1-beta, IL-10, IL-4, and IL-8). The total antioxidant capacity (TAC) increased significantly in the supplemented patients, with no significant changes observed in isoprostanes.

**Conclusion:**

The specific ONS improved protein-calorie intake, nutritional status (mainly FFM), and some biomarkers of inflammation/oxidation. The addition of probiotics could have a synergistic effect with ONS in such biomarkers.

**Clinical trail registration:**

https://clinicaltrials.gov/ct2/show/, identifier NCT03924089.

## 1. Introduction

Malnutrition, or protein-energy wasting (PEW), is highly prevalent among patients with chronic kidney disease (CKD), especially those undergoing hemodialysis, and is associated with significant morbidity and mortality. The etiology of malnutrition is multifactorial and includes decreased protein-calorie intake due to anorexia and dietary restrictions, inflammation, hypercatabolism, protein loss during dialysis, metabolic acidosis, uremic toxicity, and the presence of comorbidities (1–4). For this reason, it is recommended to evaluate periodically the appetite, dietary intake, and biochemical data, as well as to carry out a nutritional and functional (morphofunctional) assessment and an individualized approach to the diet of patients by expert professionals. When dietary advice is insufficient to achieve protein-calorie intake goals, the use of oral nutritional supplements (ONSs) is the next step to prevent and/or treat malnutrition (1, 3). The use of ONS (standard or specific for CKD) in patients with hemodialysis has shown, in some randomized studies (compared to usual follow-up), that it can increase protein-calorie intake, weight, fat-free mass (FFM), and fat and albumin concentrations, without raising the levels of electrolytes such as phosphorus or potassium (1–9). Furthermore, in retrospective studies, it has been observed that it could reduce hospital admissions and even mortality (10–12). In these patients, mechanisms of inflammation coexist with oxidative stress, which favors cardiovascular complications that could be attenuated with dietary and pharmacological interventions (13).

The Mediterranean diet has been proposed as the dietary pattern of choice for patients with CKD, which could improve endothelial function, inflammation, oxidative stress, and lipid profile, as well as reduce cardiovascular disease incidence (1, 4, 14). Some of its essential components are virgin olive oil, fish (as a source of n-3 fatty acids), and fiber from plant foods. Virgin olive oil is rich in polyphenols, and its consumption has been associated with a decrease in cardiovascular events, diabetes, and other chronic diseases (15). In hemodialysis patients with CKD, the minor polar compounds of extra virgin olive oil seem to exert an antioxidant and anti-inflammatory effect (16). The use of n-3 fatty acid supplements in patients with CKD could decrease markers of inflammation and oxidation (associated with lean mass depletion) (17). The metabolic alterations inherent to uremia and the intake of a Western-type diet could promote intestinal dysbiosis among patients with CKD and may play a key role in disease progression and complications. Dietary patterns such as the Mediterranean could reduce inflammatory processes, including leaky gut and subsequent endotoxemia (18).

In addition, the use of pre- and pro-biotics could have a crucial role in the regulation of the immune system and prevent infectious complications, treat hyperphosphatemia, reduce the levels of solutes that contribute to the uremic syndrome, as well as improve the lipid profile, oxidative stress, and systemic inflammation (19). Although some studies have shown some beneficial results, there is no conclusive rationale for recommending biotic supplements for improving outcomes in patients with CKD (20).

Whey proteins are rich in branched-chain amino acids, leucine, glutamine, and cysteine and are quickly digested; moreover, they favor a greater protein anabolic response than other protein sources. Although this requires still further evidence (21), its use in hemodialysis patients could reduce inflammatory parameters and improve physical function (22–24).

Recently, a new ONS was developed in Spain specifically designed for malnourished (or at risk) hemodialysis patients with a “similar to the Mediterranean diet” pattern (made up of functional nutrients such as extra virgin olive oil, n-3 fatty acids, whey protein, fiber, and antioxidants) that could improve the achievement of dietary as well as nutritional and functional status goals, metabolic changes, and associated inflammation and oxidative stress.

We aimed to study whether the new ONS, associated with probiotics or not, may improve nutritional and functional status and reduce biomarkers of inflammation and oxidation in malnourished hemodialysis patients, compared to individualized diet recommendations.

## 2. Materials and methods

### 2.1. Design

Randomized, multicenter, parallel-group trial with three groups, open regarding the intake of ONS or individualized diet recommendations but double-blind for the intake of probiotics. Patients were randomized to one of the following three groups (using a computer-generated random number table):

1: Control (C): received individualized dietary recommendations.
2: ONS + placebo (SU-PL): received ONS + dietary recommendations.
3: ONS + probiotics (SU-PR): received ONS with probiotics + dietary recommendations.

The Renacare^®^ ONS was specifically developed for malnourished hemodialysis patients. It is high in energy (2 kcal/ml) and proteins and enriched with functional nutrients (extra virgin olive oil, omega-3 fatty acids, whey protein, antioxidants, low-glycemic index carbohydrates, fiber, and carnitine). Table 1 shows its composition. The supplement is presented in vanilla flavor, but it includes six additional flavors that can be added to facilitate compliance, acceptance, and individualization.^1^

**TABLE 1:** Nutritional composition of the oral nutritional supplement renacare^®^.

	Units	100 ml	200 ml
Energy value	Kcal/kj	200/837	400/1674
Fats, of which	g	8.7	17
Saturated fatty acids	g	1.6	3.1
Monounsaturated fatty acids	g	5.4	11
Polyunsaturated fatty acids	g	1.8	3.6
Eicosapentaenoic acid (EPA)	mg	231	462
Docosahexaenoic acid (DHA)	mg	144	288
Carbohydrates, of which	g	20.5	41.0
Sugars	g	1.2	2.4
Dietary fiber	g	2.00	4.00
Proteins	g	8.97	17.9
Salt	g	0.15	0.30
**Vitamins**			
Vitamin A	μg-RE	50	100
Vitamin D	μg	1.35	2.70
Vitamin K	μg	9.8	19.6
Vitamin C	mg	10.0	20
Vitamin B1	mg	0.45	0.90
Vitamin B2	mg	0.50	1.00
Vitamin B6	mg	0.80	1.60
Niacin	mg-NE	4.00	8.00
Folic acid	μg	100	200
Vitamin B12	μg	1.20	2.40
Pantothenic acid	mg	0.85	1.70
Biotin	μg	3.90	7.8
Vitamin E	mg-αTE	3.50	7.00
**Minerals**			
Sodium	mg	60	120
Chloride	mg	90	180
Potassium	mg	75	150
Calcium	mg	100	200
Phosphorus	mg	30	60
Magnesium	mg	10.0	20.0
Iron	mg	2.00	4.00
Zinc	mg	2.00	4.00
Copper	μg	200	400
Iodine	μg	16.0	32.0
Selenium	μg	7.50	15.0
Manganese	mg	0.28	0.56
Chrome	μg	5.00	10.0
Molybdenum	μg	5.0	10.0
**Others**			
Coline	mg	42.0	84.0
L-carnitine	mg	120	240
Osmolarity	mOsmol/l	390	
**Ingredients**			
	**%**		
**Carbohydrates**			
Low glycemic index maltodextrin	60		
Low glycemic index dextrin	40		
**Protein**			
Whey	55		
Casein	45		
**Fats**			
Extra virgin olive oil	75		
Rapeseed oil	10		
Fish oil	12		
Others (lecithin)	3		
**Fiber**			
FOS	35		
Acacia	35		
Oat fiber	30		

FOS, fructooligosaccharides. https://adventiapharma.com/nutricion-clinica/productos/enteral-oral/bi1-renacare-dialysis/.

The individualized nutritional requirements of all patients were estimated based on the recommendations of the International Society of Renal Nutrition and Metabolism. Protein intake targets were more than 1.2 g/kg/day (3). All participants had face-to-face interviews with a dietitian at baseline and after 3 and 6 months. Patients randomized to the ONS groups were recommended to ingest two bricks per day (400 ml) [with a minimum of one daily (200 ml)]. The daily intake of ONS was prospectively recorded in a data collection sheet by the patients. The probiotics and the placebo were supplied in capsules completely indistinguishable by their external appearance (one capsule of 380 g). Each capsule of probiotic contained live bacteria: *Bifidobacterium breve* CNCM I-4035 [1.00E + 09 colony forming units (CFU)], *Bifidobacterium animalis lactis* BPL1 CECT 8145 (3.50E + 09 CFU), and *Lactobacillus paracasei* CNCM I-4034 (5.00E + 08 CFU).

The Research Ethics Committee provincial of Málaga approved the study, and the protocol meets the Ethical Standards of the Declaration of Helsinki. The study was registered with the following code: NCT03924089.

### 2.2. Inclusion and exclusion criteria

Inclusion criteria comprised adult subjects (> 18 years) undergoing hemodialysis for more than 6 months before inclusion and at least one of the following malnutrition criteria: (a) involuntary weight loss > 5% in 3 months or > 10% in 6 months; (b) serum albumin < 3.5 g/dl or prealbumin < 28 mg/dl; (c) body mass index (BMI) < 23 kg/m^2^; (d) muscular mass loss > 5% in 3 months or > 10% in 6 months; and (e) low muscle mass or strength: FFM index (FFMI) lower than 15 kg/m^2^ in women or lower than 17 in men or Jamar hand dynamometry in the dominant arm (maximum or mean of three determinations) lower than the fifth percentile of the Spanish population (25). Standard hemodialysis therapy (3 days/week, 240 min, high-flux dialyzer, blood flow > 250 ml/min, and dialysate with bicarbonate buffer with a flow 500 ml/min; Kt/V 1.3) or online hemodiafiltration with high reinfusion rate therapy not being modified in the 3 months before inclusion. Written informed consent was obtained.

Exclusion criteria were not signing the informed consent, type 1 diabetes mellitus or type 2 diabetes mellitus with glycated hemoglobin > 9%, unstable dry weight, limb amputation, significant edema, active malignancy, hospital admissions in the last 3 months, acute gastrointestinal disease in the 2 weeks before the inclusion, gastrectomy, gastroparesis or abnormal gastric emptying, heart failure grade IV, severe hepatic insufficiency, alcohol or other drugs abuse, participants enrolled in another research study at inclusion, pregnant women, patients who received any ONS in the 4 weeks before the inclusion, receiving enteral tube feeding, galactosemia, fructosemia, or requirement of a no-fiber diet, allergy or hypersensitivity to any ingredient of the ONS, ongoing treatment with glucocorticoids, oral fatty acids omega-3 supplement in the last 4 weeks before inclusion, intradialytic parenteral nutrition in the last 3 months prior to inclusion, or having received any pro- or prebiotics (not as part of the diet) in the last 3 months before inclusion.

### 2.3. Outcomes

Examinations were performed at baseline and after 3 and 6 months.

#### 2.3.1. Dietary questionnaire

A 5-day prospective dietary questionnaire (including one weekend day) was fulfilled. The data were analyzed using a computer application designed by our group for this purpose (Dietstat^®^) (26). The composition of the ONS was also included in the database.

A 14-item dietary screening questionnaire was used to assess adherence to the Mediterranean diet. This is a self-administered validated dietary questionnaire used in the PREDIMED trial (Prevención con Dieta Mediterránea). The score ranges from 0 to 14, with higher scores representing greater adherence to the Mediterranean diet (15).

#### 2.3.2. Morphofunctional nutritional assessment

Height and weight were determined with a calibrated stadiometer and scale. Body mass index (BMI) was defined as the weight in kilograms divided by squared height (in meters). “Dry weight” was measured 30 min after the end of dialysis. Mid-arm circumference was obtained with an inextensible tape measure. Skinfold thickness (tricipital) measurements were conducted using a constant pressure lipocalibrator (Holtain Limited) by the same researcher in each hospital. Three measurements were completed, and values were averaged. Mid-arm muscle circumference was calculated as mid-arm circumference minus π times triceps skinfold thickness. Bioelectrical impedance analysis was performed using a tetrapolar 50-kHz bioelectrical impedance analyzer (BIA 101 RJL, Akern Bioresearch, Firenze, Italy). FFMI was calculated (FFM in kg/height in m^2^).

Muscle strength was assessed using a dynamometer (Jamar handgrip; Asimow Engineering Co., Los Angeles, CA, USA) prior to the start of dialysis in the dominant hand, this was repeated on three occasions, and the mean was recorded.

The patients performed, prior to the start of dialysis, the short physical performance battery (SPPB) test (consisted of gait speed, a sit-to-stand test performed five times, and balance tests) and was calculated using the previously defined methods (scores ranged between 0 and 12) (27).

#### 2.3.3. Biomarkers

Fasting blood samples were drawn before beginning the dialysis session; plasma and serum were separated into aliquots and stored until analysis at −80°C in the Hospital-IBIMA biobank. One aliquot was analyzed immediately in an autoanalyzer at the laboratories of each hospital to measure C-reactive protein (CRP), triglycerides, cholesterol, creatinine, urea, electrolytes, blood liver function, albumin, prealbumin, and glycated hemoglobin. Vitamin D was analyzed by electrochemiluminescent immunoassay (Modular E-170, Roche Diagnostics). The serum levels of antioxidant biomarkers were determined by enzyme immunoassay techniques following the manufacturer’s instructions in Versamax (MTX Lab System, Barcelona, Spain): Cayman’s Antioxidant Assay (CAT) (Cayman Chemical Company, MI, USA; Intra-Assay CV = 3.4%; Inter-Assay CV = 3%), 8-isoprostane (Cayman Chemical Company, MI, USA; Intra-Assay CV = 7.6–12%; Inter-Assay CV = 9.7–19.9%). Proinflammatory cytokines and atherosclerosis biomarkers [brain-derived neurotrophic factor (BDNF), bone morphogenetic protein-2 (BMP-2), CD62E (E-selectin), interferon-gamma, interleukin (IL)-1-alpha, IL-1-beta, IL-10, IL-12p70, IL-13, IL-15, IL-17A (CTLA-B), interleukin-1 receptor antagonist, IL-4, IL-6, IL-8 (CXCL8), cytokine-leukemia inhibitory factor (LIF), and monocyte chemoattractant protein-1 (MCP-1), TNF-alpha] were measured in 25 ul of serum with ProcartaPlex Multiplex Immunoassay (Thermo Fisher Scientific, Waltham, MA, USA) following manufacturer’s instructions. For VCAM-1 and ICAM-1, we have diluted the sample 100 times. All measurements were performed in duplicate, and the serum concentrations were obtained with a standard curve.

#### 2.3.4. Adherence and side effects

At each visit, the patients filled out questionnaires to assess the presence and intensity of gastrointestinal symptoms in the 30 days prior to the visit on a scale from 0 to 10 (nausea, vomiting, diarrhea, constipation, reflux, pain, and bloating). In addition to the scheduled visits, the research team made weekly phone calls during the first month and subsequently every 15 days until the end of the study to detect the presence of adverse effects and encourage adherence to diet, supplementation, probiotics, and exercise. A survey was conducted on the acceptance of the supplement and its organoleptic characteristics at months 3 and 6. All patients received individualized physical exercise recommendations based on their SPPB scores.

### 2.4. Statistical analysis

Data analysis was conducted using the IBM SPSS Statistics Version 26 (IBM Corp. Released 2019. IBM SPSS Statistics for Windows, Version 26.0. Armonk, NY: IBM Corp.) program. Quantitative variables were expressed as mean ± SD or median and interquartile range according to normality. In the case of cytokines, log transformation was applied. Normality was assessed by Shapiro–Wilk test.

For the analysis of socio-demographic and basal-clinical characteristics, the chi-square test with Fisher’s exact distribution was used for qualitative variables; whereas for quantitative variables, the ANOVA test for independent variables or the Kruskal–Wallis *H*-test was used, according to normality. To compare variables according to the group of study and the modifications along time (at baseline, 3 and 6 months), ANOVA for repeated variables was used, if applicable. Otherwise, the necessary non-parametric techniques were applied: intra-subject Friedman (*post hoc* Wilcoxon) and inter-subject Kruskall–Walis *H* (*post hoc* Mann–Whitney *U*). The level of significance taken into account was 5%; for multiple comparisons (*post hoc*), Bonferroni correction was considered. Data were analyzed as the intention to treat.

The sample size was estimated according to changes in albumin levels in patients on hemodialysis who had received supplements vs. standard treatment (9). Assuming a (bilateral) confidence level of 95% and a potency of 80% to detect differences of at least 0.25 g/dl in albumin concentrations between the C group vs. ONS groups, and with a standard deviation of 0.25 g/dl, it was estimated to treat 17 patients per arm. To prevent 30% dropouts, it was decided to increase the sample to 22 patients per arm (total: 66 patients).

## 3. Results

**FIGURE 1:**
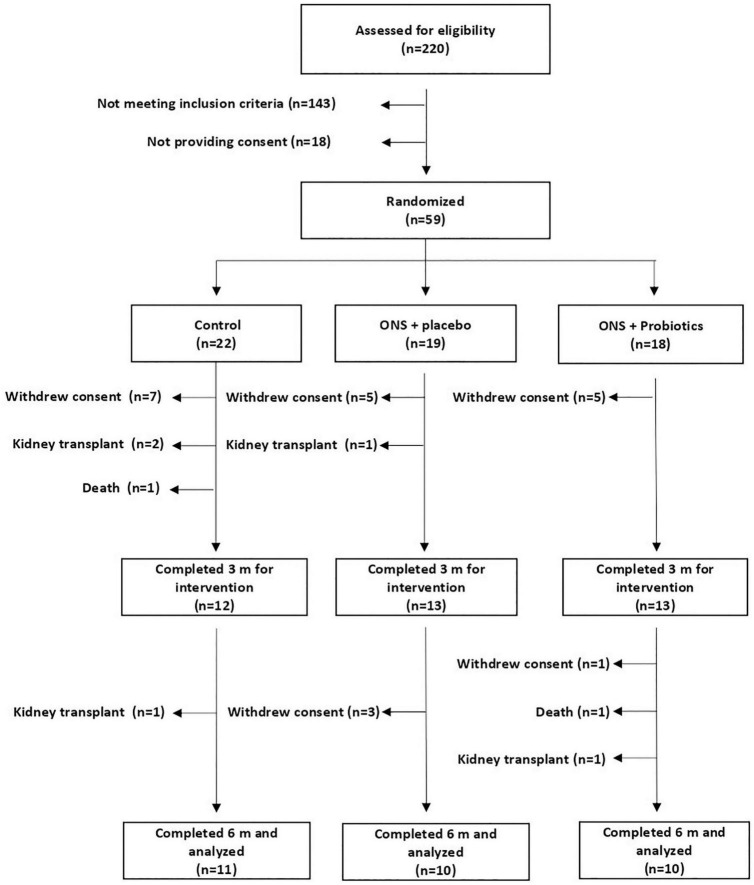
Study flowchart. ONS, oral nutritional supplement.

From the 220 subjects assessed for eligibility, 59 were randomized. Notably, 31 patients completed the 6-months trial and were analyzed (11 C group, 10 SU-PL, and 10 SU-PR) (Figure 1). The causes for consent withdrawal were in the C group, not wanting to continue with visits and individualized diet (*n* = 4), difficulties in follow-up due to the SARS-CoV-2 pandemic (*n* = 2), and the decision of their nephrologist (*n* = 1); in the SU-PL group, transfer to another facility (*n* = 1), not wanting to continue with visits (*n* = 2), difficulties in follow-up due to the SARS-CoV-2 pandemic (*n* = 2), and lack of supplement acceptance (*n* = 3); and in the SU-PR group, not wanting to continue with visits (*n* = 2), difficulties in follow-up due to the SARS-CoV-2 pandemic (*n* = 1), and lack of supplement acceptance (*n* = 3). The mean intake of supplements during the 6 months of follow-up was 1.5 ± 0.46 in the SU-PL group and 1.55 ± 0.41 in the SU-PR group. There were no significant differences in the digestive symptoms scale between groups (C, SUP-PL, SUP-PR), neither at baseline nor at any of the 6 months of follow-up (Supplementary Table 1). Supplement acceptance was high (Supplementary Table 2). There was no patient withdrawal due to gastrointestinal side effects. We observed no significant changes regarding gastrointestinal symptoms in patients quitting the study due to consent withdrawal or severe adverse effects (death, admission for transplant) and those who continued in the trial.

There were no basal significant differences between groups regarding age, sex, diabetes, Charlson comorbidity index, or intake of fermented milk or antibiotics during the month prior to inclusion (Table 2). Moreover, there were no baseline differences in any of the parameters for the morphofunctional nutritional assessment, dietary intake, biochemical data, or analyzed biomarkers (except for IL-12) (Tables 3–6).

**TABLE 2:** Baseline characteristics according to the intervention arm.

	Control (*n* = 11)	SU-PL (*n* = 10)	SU-PR (*n* = 10)	*P*-values
Age (years) m ± ds	76.3 ± 8.7	65.1 ± 18.4	66 ± 18.5	0.234
Sex, women% (n)	27 (3)	20 (2)	30 (3)	0.999
Diabetes mellitus% (n)	36.4 (4)	40 (4)	30 (3)	0.999
Antibiotic treatment in the last month	9.1 (1)	20 (2)	10 (1)	0.825
Consumption of yogurt or fermented milk in the last month% (n)	63.6 (7)	60 (6)	60 (6)	0.999
Charlson comorbidity Index. m ± ds	4 ± 2.31	5.1 ± 2.02	4.18 ± 2.6	0.262

Data are presented as mean ± standard deviation (m ± ds) or in percentage % (n), SU-PL, supplement group + placebo; SU-PR, supplement group + probiotics.

**TABLE 3:** Dietary survey.

	Basal				3 months				6 months			
	**Control *n* = 11**	**SU-PL *n* = 10**	**SU-PR *n* = 10**	**SU-TOT *n* = 20**	**Control *n* = 11**	**SU-PL *n* = 10**	**SU-PR *n* = 10**	**SU-TOT *n* = 20**	**Control *n* = 11**	**SU-PL *n* = 10**	**SU-PR *n* = 10**	**SU-TOT *n* = 20**
Energy (kcal) m ± ds	1804 ± 800	1590 ± 347	1731 ± 595	1673 ± 499	1595 ± 436	1746 ± 477	2054 ± 485*	1927 ± 492$	1577 ± 369	1938 ± 490$	1934 ± 387	1939 ± 417*@
Energy/kg (kcal/kg) m ± ds	25 ± 10	26 ± 7	27 ± 12	26 ± 10	23 ± 6	27 ± 9	31 ± 13	29 ± 11	23 ± 8	29 ± 8	28 ± 10	28 ± 9
Protein (g) m ± ds	72 ± 24	69 ± 16	70 ± 17	70 ± 19	67 ± 20	79 ± 14	83 ± 18*	77 ± 19*	69 ± 18	97 ± 24@&	83 ± 18	82 ± 22@&
Protein/kg (g/kg) m ± ds	1 ± 0.3	1.1 ± 0.3	1.1 ± 0.4	1.1 ± 0.3	1 ± 0.4	1.3 ± 0.4	1.2 ± 0.5	1.2 ± 0.4@	1 ± 0.4	1.4 ± 0.4*	1.2 ± 0.4	1.3 ± 0.4@
Total fat (g) m ± ds	74 ± 23	61 ± 17	73 ± 18	68 ± 18	68 ± 15	77 ± 26*	87 ± 22*	83 ± 23$	64 ± 14	83 ± 27*	80 ± 13	82 ± 20*@
Total fat/kg (g/kg) m ± ds	1.05 ± 0.33	1 ± 0.36	1.13 ± 0.5	1.07 ± 0.4	0.96 ± 0.2	1.21 ± 0.5*	1.28 ± 0.5	1.25 ± 0.5*@	0.93 ± 0.3	1.25 ± 0.4*	1.15 ± 0.4	1.19 ± 0.4
SF% m ± ds	29.3 ± 4.8	29.8 ± 5	30.1 ± 7	29.9 ± 5.9	26.5 ± 3.4	24.6 ± 6.2	27.9 ± 9.4	26.4 ± 7.9	29.7 ± 6.4	25.5 ± 4.4	28.6 ± 8.7	27.1 ± 7
MF% m ± ds	54.1 ± 5.9	52.2 ± 6.6	55.2 ± 6.4	54.8 ± 6.5	57.9 ± 4.5	53.5 ± 10.6	53.6 ± 8.4	53.6 ± 9.1	56.7 ± 6.4	58.8 ± 6.2	55.5 ± 8	57.0 ± 7.2
PF% m ± ds;	16.6 ± 4.3	18.0 ± 5.3	14.8 ± 3.2	16.4 ± 4.3	15.6 ± 3.8	21.9 ± 10.7	18 ± 4.2	18.2 ± 6	13.6 ± 1.7	15.7 ± 5.1	16 ± 2.8	15.0 ± 3.3
Carbohydrate (gr) m ± ds	186 ± 75	190 ± 66	198 ± 102	195 ± 87	175 ± 56	184 ± 58	221 ± 66	206 ± 64	174 ± 54	200 ± 66	217 ± 61	210 ± 62
Carbohydrate/kg (g/kg) m ± ds	2.62 ± 1	3.03 ± 1.1	3.03 ± 1.8	3.03 ± 1.5	2.5 ± 0.8	2.86 ± 0.9	3.36 ± 1.7	3.15 ± 1.5	2.54 ± 1	2.98 ± 1	3.15 ± 1.3	3.08 ± 1.2
GI m ± ds	55 ± 5	54 ± 11	47 ± 24	50 ± 19	61 ± 4	44 ± 9@	42 ± 15#	43 ± 12ß	61 ± 6	47 ± 8	44 ± 17@	45 ± 14#
GL m ± ds	103 ± 41	111 ± 42	108.4 ± 83.3	110 ± 68	105 ± 37	77 ± 19	100 ± 32	91 ± 29	105 ± 32	95 ± 43	100 ± 42	98 ± 41
Fiber (g) med (p25; p75)	11 (9; 24)	11 (8; 19)	12 (7; 16)	11 (8; 16)	10 (10; 17)	14 (11; 20)	15 (13; 17)	14 (13; 17)	14 (10; 17)	15 (14; 23)*	17 (15; 20)	17 (15; 20)$
n-3 (g) med (p25; p75)	0.78 (0.57; 1.03)	0.8 (0.6; 1.23)	0.77 (0.55; 0.95)	0.79 (0.57; 1)	1.09 (0.49; 1.22)	1.74 (0.91; 2.73)*	2.14 (1.16; 2.23)@&	1.96 (1.08; 2.25)@&	0.56 (0.45; 1.03)	1.4 (0.87; 2.87)@	1.3 (0.98; 1.63)&	1.33 (0.94; 1.74)#*
EPA (g) med (p25; p75)	0.05 (0.01; 0.13)	0.02 (0.01; 0.04)	0.03 (0.01; 0.08)	0.03 (0.01; 0.07)	0.04 (0.01; 0.21)	0.56 (0.06; 0.93)	0.86 (0.47; 1)#	0.66 (0.47; 0.97)ß+	0.04 (0.01; 0.14)	0.47 (0.02; 0.93)	0.5 (0.46; 0.8)@&	0.49 (0.28; 0.87)#*
DHA (g) med (p25; p75)	0.1 (0.04; 0.18)	0.03 (0.01; 0.11)	0.11 (0.03; 0.21)	0.06 (0.01; 0.18)	0.11 (0.04; 0.3)	0.4 (0.11; 0.58)@*	0.58 (0.29; 0.72)	0.42 (0.24; 0.67)Ω#	0.1 (0.01; 0.3)	0.29 (0.03; 0.58)	0.47 (0.29; 0.58)	0.39 (0.23; 0.58)@
K (mg) m ± ds	2164 ± 1060	2155 ± 1035	1747 ± 710	1915 ± 853	1783 ± 517	1880 ± 237	1583 ± 475	1705 ± 413	1764 ± 350	2416 ± 615	1790 ± 616	2048 ± 675
P (mg) m ± ds	1098 ± 433	1017 ± 316	1056 ± 328	1040 ± 314	1009 ± 306	1042 ± 212	1089 ± 255	1070 ± 232	1047 ± 311	1188 ± 341	1030 ± 323	1095 ± 330
Ca (mg) m ± ds	592 ± 276	612 ± 220	635 ± 332	625 ± 283	520 ± 100	741 ± 357	903 ± 356	836 ± 355	654 ± 260	793 ± 371	734 ± 260	758 ± 301
PREDIMED (total score) m ± ds	7.2 ± 1.4	7.5 ± 1.4	6.9 ± 2.3	7.2 ± 1.9	8.8 ± 1.6$	8.2 ± 1.7	7.6 ± 2.3	7.9 ± 2	8.2 ± 1.6	8.7 ± 1.5*	8 ± 1.9*	8.4 ± 1.7$

Data are presented as mean ± standard deviation (m ± ds) or median and interquartile range: med (p25; p75); SU-PL supplement group + placebo; SU-PR supplement group+ probiotics; SU-TOT: supplement group (SU-PL+SU-PR). Differences between baseline and 3 m or 6 m intragroup: *p < 0.05; $*p* < 0.01; &*p* < 0.001; differences between 3 and 6 months: €*p* < 0.05; Ω*p* < 0.01; + *p* < 0.001; Differences with respect to the control group (at baseline, 3 months, or 6 months): *p* < 0.05; #*p* < 0.01; ß*p* < 0.001. Kcal, kilocalories; g, gram; SF, saturated fat; MF, Monounsaturated fat; PF, Polyunsatured fat; GI, glycemix index; GL, glycemix load; n-3, omega-3 fatty acids; EPA, eicosapentaenoic acid; DHA, docosahexaenoic acid; K, potassium; P, phosphorus; Ca, calcium. Score PREDIMED: “Prevención con Dieta Mediterránea”.

**TABLE 4:** Morphofunctional nutritional assessment.

	Basal				3 months				6 months			
	**Control *n* = 11**	**SU-PL *n* = 10**	**SU-PR *n* = 10**	**SU-TOT *n* = 20**	**Control *n* = 11**	**SU-PL *n* = 10**	**SU-PR *n* = 10**	**SU-TOT *n* = 20**	**Control *n* = 11**	**SU-PL *n* = 10**	**SU-PR *n* = 10**	**SU-TOT *n* = 20**
Weight (kg) m ± ds	71.7 ± 11	63.2 ± 12.1	70.5 ± 17.9	66.9 ± 15.3	71 ± 9.8	64.2 ± 12.4	72.7 ± 18.7*	68.47 ± 16.05*	71.1 ± 8.8	66.3 ± 11.2*€	73.8 ± 18.5*	70 ± 15.4&€
Dry weight (kg) m ± ds	70.2 ± 10.7	61.9 ± 12.3	68.2 ± 17.5	65 ± 15.1	69.8 ± 9.6	62.9 ± 12.3	69.9 ± 18.5*	66.4 ± 15.7$	69.4 ± 9	64.4 ± 11.4*€	70.9 ± 18.1*	67.6 ± 15.1&€
FFM (kg) m ± ds	48.6 ± 7.9	45.9 ± 6.5	50.7 ± 12.2	48.3 ± 9.8	48.2 ± 7	46.2 ± 7	51.92 ± 13	49.06 ± 10.6	49.63 ± 7.8	48.11 ± 7.2	52.94 ± 12.3	50.53 ± 10.1*€
Dry FFM (kg) m ± ds	47.7 ± 7.6	45 ± 7.6	49.4 ± 12.1	47.2 ± 9.8	47.4 ± 6.9	45.1 ± 7	49.85 ± 12.8	47.5 ± 10.3	48.5 ± 8.3	46.4 ± 7	50.9 ± 12.1	48.7 ± 9.9*€
FM (kg) m ± ds	23.1 ± 7.1	17.2 ± 7.6	19.8 ± 8.4	18.5 ± 7.9	22.8 ± 6.4	18.2 ± 7.2	20.8 ± 7.4	19.5 ± 7.2	20.8 ± 6.5	18.5 ± 7.2	20.7 ± 8.7	19.6 ± 7.8
Dry FM (kg) m ± ds	22.6 ± 7	16.9 ± 7.5	18.8 ± 7.3	17.8 ± 7.3	22.4 ± 6.3	17.8 ± 7.1	20 ± 7.2	18.9 ± 7.1	20.9 ± 7.1	17.9 ± 7.3	19.9 ± 8.4	18.9 ± 7.7
TS (mm) m ± ds	15.5 ± 8.9	11.5 ± 5.7	12.4 ± 7	11.9 ± 6.2	15.2 ± 7.5	12.4 ± 5.3	13.1 ± 5.7	12.7 ± 5.4	14.2 ± 8.8	12.6 ± 6.1	13 ± 7.6	12.8 ± 6.7
FFMI (kg/m^2^) m ± ds	16.9 ± 1.3	17 ± 2.2	18 ± 3.4	17.6 ± 2.8	16.8 ± 1.1	16.4 ± 3.2	18.5 ± 3.7	17.4 ± 3.5	17.3 ± 1.5	17.8 ± 2.2	18.9 ± 3.4	18.4 ± 2.8*€
Dry FFMI (kg/m^2^) m ± ds	16.6 ± 1.3	16.6 ± 2.3	17.6 ± 3.4	17.1 ± 2.9	16.5 ± 1.1	16.7 ± 2.3	17.8 ± 3.7	17.2 ± 3	16.8 ± 1.6	17.2 ± 2.4	18.2 ± 3.3	17.7 ± 2.9*€
MUAC (cm) m ± ds	27.3 ± 2.9	26 ± 3.5	25.6 ± 4.1	25.8 ± 3.7	27.3 ± 2.6	26.1 ± 3.7	26.7 ± 4.4	26.4 ± 4	26.8 ± 2.4	26.4 ± 4.3	26.4 ± 4.5	26.4 ± 4.3
MUAMC m ± ds	22.9 ± 3.3	22.6 ± 2.9	21.6 ± 3.6	22.1 ± 3.2	22.6 ± 2.7	22.1 ± 2.6	22.6 ± 3.4	22.4 ± 2.9	23.2 ± 2.8	22.8 ± 2.9	22.3 ± 3.7	22.6 ± 3.3
HGS (kg) m ± ds	23.9 ± 9.7	17.7 ± 7.9	17.6 ± 7.1	17.7 ± 7.3	24.4 ± 10.1	20.9 ± 6.4	17.7 ± 6	19.2 ± 6.2	21.1 ± 12.1	23.7 ± 6.5*	17.9 ± 8.2	20.6 ± 7.8
SPPB (total score) m ± ds	6.9 ± 3.4	4.7 ± 3.1	7.1 ± 3.4	5.9 ± 3.4	6.8 ± 2.9	5.4 ± 3.8	6.6 ± 3.7	6 ± 3.4	7.1 ± 3.3	5.4 ± 4	6.9 ± 2.7	6.2 ± 3.4

Data are presented as mean ± standard deviation (m ± ds) or median and interquartile range: med (p25; p75); SU-PL, supplement group + placebo; SU-PR, supplement group + probiotics; SU-TOT, supplement group (SU-PL + SU-PR). Differences between baseline and 3- or 6-months intragroup: *p < 0.05; $*p* < 0.01; &*p* < 0.001; differences between 3 and 6 months: €*p* < 0.05. FFM, fat-free mass; FM, fat mass; TS, triceps skinfold; FFMI, fat-free mass index; MUAC, mid-upper arm circumference; MUAMC, mid-upper arm muscle circumference; HGS, handgrip strength.

**TABLE 5:** Blood test parameters.

	Basal				3 months				6 months			
	**Control *n* = 11**	**SU-PL *n* = 10**	**SU-PR *n* = 10**	**SU-TOT *n* = 20**	**Control *n* = 11**	**SU-PL *n* = 10**	**SU-PR *n* = 10**	**SU-TOT *n* = 20**	**Control *n* = 11**	**SU-PL *n* = 10**	**SU-PR *n* = 10**	**SU-TOT *n* = 20**
K (mEq/L) m ± ds	4.9 ± 0.6	5.3 ± 0.7	5.4 ± 0.7	5.3 ± 0.7	5.1 ± 0.6	5.3 ± 0.9	5 ± 0.8	5.1 ± 0.9	4.9 ± 0.68	4.9 ± 0.57	4.6 ± 0.6	4.8 ± 0.6
P (mg/dL) m ± ds	4.6 ± 0.8	4.1 ± 0.9	4.1 ± 0.7	4.1 ± 0.8	4.5 ± 1.2	4.2 ± 1.5	3.6 ± 1.2	3.9 ± 1.4	4 ± 1.2	4.4 ± 1.3	3.7 ± 1.4	4.1 ± 1.3
Ca (mg/dL) m ± ds	8.9 ± 0.6	8.6 ± 1.5	9 ± 0.7	8.8 ± 1.2	9 ± 0.6	9.3 ± 0.6	8.9 ± 0.9	9.1 ± 0.8	9.3 ± 0.5	8.9 ± 0.6	9 ± 0.9	8.9 ± 0.8
Creatinine (mg/dL) m ± ds	7.3 ± 32	7.4 ± 1.4	6.1 ± 1.3	6.9 ± 1.5	7.1 ± 1.	7.5 ± 2.2	6.6 ± 0.9	7.1 ± 1.8	6.9 ± 1.5	7.9 ± 2.4	6.2 ± 1.5	7.1 ± 1.8
Urea (mg/dl) m ± ds	110.1 ± 32.2	118.7 ± 30.1	91 ± 25.9	107.6 ± 30.8	113.1 ± 25.5	127.7 ± 40.6	113.6 ± 30.6	118.7 ± 32.8	108.8 ± 23.02	150.1 ± 41.4*	88.7 ± 28.4	118.1 ± 40.7ω
Uric acid (mg/dL) m ± ds	5.5 ± 1.6	6.2 ± 1.5	5.3 ± 1.9	5.8 ± 1.7	4.5 ± 0.3	6 ± 1.4	5.7 ± 1.6	5.9 ± 1.4	4.4 ± 0.8	6.5 ± 2.1	5.1 ± 1	5.9 ± 1.8
Total cholesterol (mg/dL) m ± ds	138.6 ± 28.9	116.1 ± 28.5	138.3 ± 30.7	125.8 ± 30.6	132.1 ± 31.6	132.4 ± 34*	125 ± 25	129.2 ± 29.6	127.8 ± 20.5	116.2 ± 38.8	140.1 ± 20.9	126.7 ± 33.5
LDL cholesterol (mg/dL) m ± ds	72.5 ± 23.8	60.8 ± 19.9	75.6 ± 36.5	68.2 ± 29.4	65.4 ± 27.4	69.6 ± 28	68 ± 40.2	68.8 ± 33.4	58.7 ± 19.4	64.4 ± 33.5	75.4 ± 32.3	69.9 ± 32.3
HDL cholesterol (mg/dL) m ± ds	44.8 ± 14.3	36.3 ± 9.3	47.1 ± 7.2	41.7 ± 9.8	46.2 ± 12.7	37.3 ± 9.5	43.6 ± 5.7*	40.4 ± 8.3	45.9 ± 12.1	36.1 ± 10.3	45.1 ± 6.1	40.6 ± 9.4
Triglycerides (mg/dL) m ± ds	106 ± 36.8	109.4 ± 33.1	92 ± 42.9	102.5 ± 36.9	104.5 ± 46.9	133.2 ± 66.8	143.2 ± 68.5*	137.2 ± 65.2	114.7 ± 59.3	106.6 ± 32.3	120.33 ± 39.6	112.1 ± 34.7
Albumin (g/dL) m ± ds	3.69 ± 0.51	3.5 ± 0.72	3.31 ± 0.42	3.41 ± 0.58	3.66 ± 0.63	3.69 ± 0.74	3.45 ± 0.49	3.57 ± 0.62	3.58 ± 0.69	3.53 ± 0.77	3.32 ± 0.6	3.43 ± 0.68
Prealbumin (mg/dL) m ± ds	25.8 ± 5.2	23.5 ± 4.6	24.2 ± 7.1	23.8 ± 5.7	24.7 ± 3.5	26.9 ± 8.7	26.6 ± 4.8	26.7 ± 6.9*	23.6 ± 3	24 ± 7.4	25 ± 7.7	24.5 ± 7.3
25-OH-vitamin D3 (ng/mL) m ± ds	24.3 ± 9.3	19.7 ± 9.6	19.7 ± 12.5	19.7 ± 11	29.7 ± 14.2	24.5 ± 11.9	30.3 ± 19.6*	27.7 ± 16.4*	25.4 ± 11.9	18.6 ± 12	23.2 ± 13	21. ± 12.4
HbA1c (%) med (p25; p75)	5.4 (4.9; 5.8)	5.2 (4.7; 5.7)	5.3 (5.15; 6.25)	5.3 (5; 5.8)	5.3 (5; 5.7)	5.5 (4.7; 5.9)	5.3 (5.1; 6.9)	5.4 (4.9; 6.3)	5.4 (5.1; 5.8)	5.3 (5.1; 5.6)	5.5 (5.2; 6.5)	5.5 (5.1; 6.3)
Hemoglobin g/dL m ± ds	11.1 ± 1.1	10.8 ± 1.1	10.8 ± 1.3	10.9 ± 1.1	11.5 ± 0.8	11.1 ± 1.0	11.3 ± 1.3	11.3 ± 1.0	11.3 ± 1.1	10.4 ± 1.1	10.8 ± 1.4	10.9 ± 1.2

Data are presented as mean ± standard deviation (m ± ds) or median and interquartile range: med (p25; p75); SU-PL, supplement group + placebo; SU-PR, supplement group + probiotics; SU-TOT, supplement group (SU-PL + SU-PR). Differences between baseline and 3- or 6-months intragroup: *p < 0.05. Differences between SU-PR and SU-PL (at baseline, 3 or 6 months): ω*p* < 0.05. K, potassium; P, phosphorus; Ca, calcium; LDL, low-density lipoprotein; HDL, high-density lipoprotein; OH, hydroxy; HbA1c, glycated hemoglobin.

**TABLE 6:** Inflammation and oxidation biomarkers.

	Basal				3 months				6 months			
	**Control *n* = 11**	**SU-PL *n* = 10**	**SU-PR *n* = 10**	**SU-TOT *n* = 20**	**Control *n* = 11**	**SU-PL *n* = 10**	**SU-PR *n* = 10**	**SU-TOT *n* = 20**	**Control *n* = 11**	**SU-PL *n* = 10**	**SU-PR *n* = 10**	**SU-TOT *n* = 20**
BDNF med (p25; p75)	1.73 (1.18; 1.9)	1.6 (1.11; 2.14)	1.6 (0.96; 2.12)	1.6 (1.04; 2.13)	1.38 (0.81; 2.01)	1.35 (1.12; 1.85)	1.29 (0.9; 2.17)*	1.35 (0.98; 2.06)	1.59 (0.05; 1.95)	1.1 (0.55; 1.87)	1.38 (0.37; 1.77)	1.29 (0.46; 1.79)*
BMP-2 med (p25; p75)	1.36 (1.19; 1.54)	1.46 (1.28; 1.65)	1.62 (1.24; 1.92)	1.56 (1.26; 1.67)	1.32 (1.12; 1.38)	1.51 (1.23; 1.9)	1.66 (1.31; 2.36)	1.57 (1.27; 2.13)	1.23 (1.12; 1.8)	1.36 (1.21; 1.64)	1.58 (1.12; 2.28)	1.43 (1.15; 1.76)*Ω
MCP-1 m ± ds	1.32 ± 0.22	1.33 ± 0.22	1.38 ± 0.39	1.36 ± 0.31	1.35 ± 0.28	1.31 ± 0.28	1.44 ± 0.42	1.38 ± 0.36	1.25 ± 0.25	1.23 ± 0.16	1.23 ± 0.17€	1.23 ± 0.16 €
CRP (mg/dL) med (p25; p75)	2.9 (0.7; 3.2)	2.9 (0.5; 10.3)	2.95 (2.9; 9.3)	2.9 (0.7; 9.6)	2.9 (0.4; 2.9)	2 (0.5; 10.7)	2.9 (0.4; 2.9)	2.9 (0.9; 10.7)	3.3 (2.2; 5.8)	2.9 (0.4; 17.3)	2.9 (2.6; 17.1)	2.9 (0.4; 17.3)
TNF-α med (p25; p75)	1.4 (1.4; 1.4)	1.41 (1.4; 1.41)	1.4 (1.4; 1.44)	1.41 (1.4; 1.43)	1.4 (1.4; 1.42)	1.4 (1.4; 1.44)	1.43 (1.4; 1.46)	1.41 (1.4; 1.46)	1.4 (1.39; 1.4)	1.4 (1.4; 1.41)	1.4 (1.4; 1.42)	1.4 (1.4; 1.42)
VCAM-1 med (p25; p75)	6.06 (5.9; 6.11)	6 (5.86; 6.23)	6.01 (5.88; 6.27)	6 (5.87; 6.23)	6.06 (5.9; 6.11)	6 (5.86; 6.23)	6.01 (5.88; 6.27)	6.12 (6.01; 6.25)	5.97 (5.91; 6.06)	6.09 (5.81; 6.16)	6.04 (5.92; 6.14)	6.07 (5.87; 6.15)
ICAM-1 med (p25; p75)	5.81 (5.65; 5.89)	5.82 (5.74; 5.99)	5.85 (5.75; 6.04)	5.82 (5.74; 6.02)	5.85 (5.65; 5.91)	5.9 (5.77; 5.99)	5.86 (5.74; 5.97)	5.88 (5.75; 5.98)	5.73 (5.6; 5.87)	5.81 (5.74; 5.94)	5.86 (5.73; 6)	5.81 (5.73; 5.97)
E-selectin med (p25; p75)	3.96 (3.9; 4.07)	4.05 (3.95; 4.24)	4.1 (3.93; 4.16)	4.09 (3.94; 4.16)	3.92 (3.81; 4.07)	4.13 (4.05; 4.17)	4.1 (3.98; 4.16)	4.11 (4.02; 4.16)	3.95 (3.77; 4)	4.08 (4.07; 4.12)	4 (3.9; 4.1)	4.08 (3.95; 4.11)
IFN-γ med (p25; p75)	0.85 (0.82; 0.87)	0.88 (0.85; 0.94)	0.87 (0.82; 0.99)	0.87 (0.85; 0.96)	0.86 (0.82; 0.9)	0.85 (0.85; 0.97)	0.91 (0.85; 1.01)	0.91 (0.85; 1.01)	0.82 (0.81; 0.85)	0.87 (0.85; 0.9)	0.86 (0.82; 0.91)	0.86 (0.82; 0.91)
IL-1 alfa med (p25; p75)	−0.54 (−0.56; −0.42	−0.41 (−0.49; −0.21)	−0.4 (−0.54; −0.14)	−0.41 (−0.51; −0.2)	−0.56 (−0.62; −0.42)	−0.31 (−0.41; −0.18)	−0.33 (−0.54; 0.25)	−0.33 (−0.49; −0.01)	−0.57 (−0.59; −0.27)	−0.38 (−0.39; −0.32)	−0.38 (−0.55; 0.13)	−0.38 (−0.54; −0.27)
IL-1β med (p25; p75)	0.15 (0.07; 0.18)	0.18 (0.18; 0.23)	0.18 (0.13; 0.27)	0.18 (0.13; 0.25)	0.13 (0.13; 0.18)	0.18 (0.13; 0.18)*	0.18 (0.13; 0.31)	0.18 (0.13; 0.27)	0.13 (0.07; 0.13)	0.13 (0.13; 0.18)	0.14 (0.13; 0.18)	0.13 (0.13; 0.18)*
IL-1RA med (p25; p75)	2.55 (2.41; 2.69)	2.37 (2.28; 2.44)	2.43 (2.3; 2.74)	2.37 (2.29; 2.68)	2.41 (2.36; 2.62)	2.5 (2.34; 2.63)	2.44 (2.36; 2.99)	2.47 (2.35; 2.77)	2.35 (2.26; 2.42)	2.4 (2.26; 2.48) €	2.48 (2.38; 2.62)	2.44 (2.27; 2.52)
IL-4 med (p25; p75)	0.66 (0.55; 0.66)	0.7 (0.66; 0.89)	0.84 (0.66; 1.29)	0.74 (0.66; 0.91)	0.66 (0.55; 0.74)	0.7 (0.66; 0.81)	0.81 (0.55; 0.92)	0.72 (0.63; 0.89)	0.55 (0.55; 0.66)	0.68 (0.66; 0.81)	0.66 (0.55; 0.66)*	0.66 (0.6; 0.74)
IL-6 med (p25; p75)	0.62 (0.46; 0.74)	0.79 (0.62; 1.08)	0.75 (0.65; 1.24)	0.77 (0.64; 1.22)	0.65 (0.35; 0.86)	0.89 (0.55; 1.01)	0.88 (0.55; 1.08)	0.88 (0.55; 1.05)	0.68 (0.46; 0.8)	0.72 (0.55; 1.04)	0.87 (0.62; 1.01)	0.77 (0.59; 1.03)
IL-8 med (p25; p75)	0.41 (0.22; 0.68)	0.39 (0.3; 0.93)	0.66 (0.51; 0.69)	0.59 (0.3; 0.93)	0.41 (0.13; 0.59)	0.47 (0.23; 0.86)	0.55 (0.3; 0.82)	0.51 (0.26; 0.86)	0.28 (0.2; 0.44)	0.35 (0.23; 0.41)	0.37 (0.27; 0.52)	0.37 (0.25; 0.52)*€
IL-10 med (p25; p75)	0.12 (0.09; 0.15)	0.17 (0.09; 0.32)	0.3 (0.18; 0.43)	0.23 (0.11; 0.4)	0.11 (0.07; 0.15)	0.17 (0.09; 0.29)	0.3# (0.15; 0.54)	0.23@ (0.12; 0.46)	0.14 (0.09; 0.17)	0.16 (0.06; 0.25)	0.27 (0.06; 0.43)	0.22 (0.06; 0.38)
IL-12 med (p25; p75)	0.89 (0.88; 0.91)	0.91 (0.89; 0.92)	0.91 (0.91; 0.99)@	0.91 (0.89; 0.93)@	0.91 (0.88; 0.91)	0.91 (0.89; 0.91)	0.92 (0.91; 0.96)	0.91 (0.89; 0.93)	0.88 (0.88; 0.89)	0.91 (0.89; 0.93)	0.91 (0.89; 0.93)	0.91 (0.89; 0.93)@
IL-13 med (p25; p75)	0.8 (0.79; 0.8)	0.79 (0.79; 0.8)	0.79 (0.79; 0.8)	0.79 (0.79; 0.8)	0.79 (0.79; 0.79)	0.79 (0.79; 0.79)	0.8 (0.79; 0.81)	0.79 (0.79; 0.8)	0.79 (0.79; 0.79)	0.79 (0.79; 0.8)	0.79 (0.79; 0.8)	0.79 (0.79; 0.8)
IL-15 med (p25; p75)	0.9 (0.88; 0.91)	0.9 (0.9; 0.91)	0.9 (0.9; 0.93)	0.9 (0.9; 0.93)	0.89 (0.88; 0.9)	0.9 (0.88; 0.92)	0.91 (0.9; 0.93)	0.9 (0.89; 0.93)	0.88 (0.87; 0.88)	0.89 (0.88; 0.9)	0.91 (0.87; 0.91)	0.9 (0.88; 0.91)
IL-17A med (p25; p75)	0.42 (0.37; 0.46)	0.44 (0.37; 0.5)	0.37 (0.32; 0.57)	0.4 (0.35; 0.53)	0.37 (0.37; 0.42)	0.4 (0.37; 0.42)	0.37 (0.32; 0.68)	0.37 (0.37; 0.55)	0.4 (0.37; 0.42)	0.4 (0.35; 0.42)	0.35 (0.35; 0.37)	0.37 (0.35; 0.42)
LIF med (p25; p75)	0.21 (0.21; 0.21)	0.19 (0.13; 0.21)	0.25 (0.13; 0.35)	0.21 (0.13; 0.35)	0.21 (0.13; 0.25)	0.25 (0.21; 0.4)	0.25 (0.13; 0.45)	0.25 (0.17; 0.42)	0.21 (0.17; 0.21)	0.23 (0.13; 0.29)	0.25 (0.21; 0.29)	0.23 (0.15; 0.29)
TAC med (p25; p75)	0.37 (0.31; 0.38)	0.33 (0.27; 0.47)	0.35 (0.26; 0.4)	0.33 (0.26; 0.47)	0.36 (0.33; 0.39)	0.46 (0.37; 0.48)	0.41 (0.31; 0.58)*	0.41 (0.31; 0.58)*	0.43 (0.33; 0.5)	0.43 (0.39; 0.48)	0.47 (0.32; 0.62)*	0.43 (0.34; 0.59)*
Isoprostanes med (p25; p75)	0.94 (0.91; 1.22)	1.17 (0.98; 1.54)	1.36 (0.81; 1.56)	1.31 (0.87; 1.56)	1.23 (0.98; 2)	1.55 (1.04; 1.89)	1.13 (0.72; 1.39)	1.34 (0.91; 1.77)	1.51 (1.16; 1.6)	1.44 (0.38; 1.64)	1.42 (0.88; 1.6)	1.42 (0.88; 1.63)

All values shown in this table come from the logarithm of the correspondent parameter. Data are presented as mean ± standard deviation (m ± ds) or median and interquartile range: med (p25; p75). SU-PL, supplement group + placebo; SU-PR, supplement group + probiotics; SU-TOT, supplement group (SU-PL + SU-PR). Differences between baseline and 3- or 6-months intragroup: *p < 0.05; differences between 3 and 6 months: €*p* < 0.05; Ω*p* < 0.01; differences with respect to the control group (at baseline, 3 or 6 months): @*p* < 0.05; #*p* < 0.01. BDNF, brain-derived neurotrophic factor; BMP-2, bone morphogenetic protein-2; MCP-1, monocyte chemoattractant protein-1; CRP, C-reactive protein; TNF-α, tumor necrosis factor-alpha; VCAM-1, vascular cell adhesion molecule-1; ICAM-1, intercellular adhesion molecule 1; IFN-γ, interferon-gamma; IL, interleukin; IL-1RA, interleukin-1 receptor antagonist; LIF, cytokine-leukemia inhibitory factor; TAC, total antioxidant capacity.

### 3.1. Dietary questionnaire

The groups that took supplements increased significantly their energy, fat (total and n-3), protein, and fiber intake compared to baseline and reached significance with regard to the C group at months 3 and/or 6 (Table 3). A significant decrease in the glycemic index in patients on ONS was observed compared to the baseline and the C group. There were no differences regarding the intake of carbohydrates, potassium, phosphorus, or calcium between groups during the intervention. The 14-point PREDIMED scale increased in all groups and for all time periods, reaching statistical significance in the C group at month 3 and supplemented groups at month 6 when compared to baseline (Table 3).

### 3.2. Morphofunctional nutritional assessment

Weight and “dry weight” increased significantly in the SU-PL, SU-PR, and SU-TOT groups at month 6, with respect to baseline and month 3. FFM, FFMI, and “Dry FFM and FFMI” increased significantly in the SU-TOT group at month 6 with respect to baseline, and at month 6 with respect to month 3. Mean hand grip strength increased significantly only in the SU-PL group at month 6, compared to baseline. Fat mass, triceps skinfold, mid-upper arm circumference, and mid-upper arm muscle circumference increased in the groups on ONS, although without reaching significance. There were no differences regarding the score in the SPPB functionality scale (Table 4).

### 3.3. Biomarkers

Prealbumin and 25-OH-Vitamin D3 levels increased significantly in the SU-TOT group at month 3 compared to baseline, and 25-OH-Vitamin D3 levels did so in SU-PR. Albumin showed a tendency toward an increase in patients on ONS that did not reach significance. Total cholesterol increased after 3 months compared to baseline in the SU-PL group, and triglycerides also at month 3 in SU-PR. We observed a decrease in potassium, especially in the SU-PR group, although it was not significant. There were no changes in phosphorus, calcium, creatinine, uric acid, and hemoglobin in any of the groups (Table 5).

Although there was a tendency toward a decrease in most parameters studied, we observed no significant differences in the inflammation markers such as CRP, ICAM-1, VCAM-1, E-selectin, IFN-gamma, IL-1-alpha, IL-12, IL-13, IL-15, IL-17, IL-1RA, LIF, IL-6, and TNF-alpha.

Brain-derived neurotrophic factor levels reached significant differences with respect to baseline at month 3 in the SU-PR group and at month 6 in SU-TOT. BMP-2 levels decreased significantly after 6 months in the SU-TOT group, compared to baseline. MCP-1 levels decreased significantly in the SU-PR and SU-TOT groups between months 3 and 6. IL-1-beta levels decreased significantly with regards to baseline at month 6 in the SU-TOT group and at month 3 in SU-PL. IL-10 levels increased in the groups on ONS at month 3, with this reaching significance in the SU-TOT and SU-PR compared to the C group. IL-4 levels decreased significantly with respect to baseline at month 6 in the SU-PR group. IL-8 decreased significantly with regard to baseline at month 6 and in the SU-TOT group between months 3 and 6.

Total antioxidant capacity (TAC) increased significantly in patients supplemented with ONS, with respect to baseline (SU-PR and SU-TOT), at months 3 and 6. Isoprostanes levels also increased, especially in the control group, although this did not reach statistical differences either over time or between groups (Table 6).

## 4. Discussion

In this study, we have observed that supplementation with a new ONS specifically designed for malnourished subjects with CKD on hemodialysis improves dietary intake and nutritional status, as well as some biomarkers of inflammation and oxidation, compared to a group on individualized treatment.

In the groups on ONS, we observed an increase in the total caloric (approximately 250 kcal mean), protein (12 g mean), fat (including n-3 fatty acids), and fiber intake, as well as a decrease in glycemic index and an improvement in adherence to the Mediterranean diet pattern. The purpose of the use of ONS should always be complementing, and never substituting, the intake of natural food. In this study, the addition of ONS to the patients’ regular diet helped to achieve the intake recommended in clinical guidelines (1). The increase in energetic intake due to the use of renal-specific protein-energy ONS has also been observed in other randomized studies in patients receiving maintenance hemodialysis (MHD) (7, 28), but not in all of them (1, 5). The new Kidney Disease Outcomes Quality Initiative (KDOQI) clinical practice guideline for nutrition in CKD recommended, for patients on MHD who are metabolically stable, prescribing a dietary protein intake of 1.0–1.2 g/kg body weight per day to maintain a stable nutritional status. Nevertheless, in malnourished (or at-risk) patients with PEW, the recommended intake could be higher (3). In previous randomized studies, no increase in protein intake due to protein-energy supplements had been found (5, 7, 29, 30), although the isolated use of some protein-based supplements did help to achieve an increase (21, 31, 32). The main protein intake in supplemented patients in this study increased to over 1.2 g per kg body weight after the intervention, which, together with the fact that half of the protein in the ONS comes from whey protein, may have contributed to the improvement in body composition we observed. The KDOKI guidelines do not routinely recommend supplementation with n-3 polyunsaturated fatty acids (PUFAs) in order to reduce the risk of mortality or cardiovascular events, although they may be prescribed at pharmacological doses to improve lipid profile. In some studies, the use of n-3 fatty acid supplements in patients with CKD seems to decrease inflammatory and oxidation markers (associated with FFM depletion) (17). In our sample, supplemented patients increased their intake of n-3 PUFA, which may have also contributed to the decrease in biomarkers of inflammation and oxidation. In patients randomized to ONS, there was also a higher intake of fiber and a lower glycemic index; moreover, the score in PREDIMED at 6 months was also higher. All these changes may have improved body composition through different anti-inflammatory and antioxidant mechanisms (4, 14, 16, 18, 33).

Malnutrition in patients with MHD is characterized by changes in body composition, especially a decrease in FFM and muscle strength, and it is associated with high morbidity and mortality (34, 35). In our study, patients taking supplements improved their body composition, with a mean increase of 2.6 kg in dry weight, from which 1.5 kg were FFM. These data are similar to those observed in other clinical trials with energy-based and/or protein-based ONS (1, 22, 29, 36). The increase in only fat mass found in other trials with ONS may not be beneficial for this population as it could increase insulin resistance and systemic inflammation (28). Tomayko et al. demonstrated, after intradialytic supplementation with whey or soy protein, improvements in gait speed and shuttle walk test performance (22); however, in the IHOPE study that combined whey protein + exercise, no significant changes compared to the control group were observed (21). In our study, the increase in FFM was associated with an improvement in hand grip strength only in the SU-PL group, and no changes in functionality, measured by SPPB, were found. Although patients were highly encouraged to perform individualized physical exercise, no supervised specific program was designed to improve the results (37).

The changes described in diet and body composition were associated with an increase in prealbumin that reached significant differences in the groups on ONS at month 3. There was also a non-significant increase in albumin. In other randomized trials comparing the use of protein or energy-protein-based ONS in patients with MHD, there were variable results, with albumin levels tending to increase moderately, especially in malnourished patients compared to control or placebo (1, 9, 36); on the contrary, there were no differences regarding prealbumin (5, 7, 38).

Serum albumin and prealbumin may be considered complementary tools to assess nutritional status and as a predictor of hospitalization and mortality; however, they are influenced by non-nutritional factors, especially the degree of inflammation (1). In our study, we have found significant decreases in several biomarkers of inflammation (BDNF, BMP-2, MCP-1, IL-1-beta, IL-4, and IL-8) in supplemented patients, especially in the group on also probiotics. Apart from being used as a biomarker of inflammation, the BDNF is increased in patients with MHD with sarcopenia and frailty (39, 40), BMP-2 is associated with increased oxidative stress and vascular risk (41), and MCP-1 has been used as a marker of structural kidney damage as well as arteriogenic factor in patients with MHD with cardiovascular disease (42–44). In contrast, there is also an increase in IL-10 levels after 3 months, which behaves as an anti-inflammatory cytokine, and whose decrease is associated with increased morbidity and reduced muscle strength in patients with MHD (45). In other randomized studies, supplementation with energy-protein-based ONS did not produce any change in CRP (7, 21, 22, 28–31, 36) or IL-6 (21, 38) levels; nevertheless, intradialytic supplementation with whey or soy protein reduced serum levels of IL-6 (22).

The SU-PR group reached significant differences over time in several biomarkers such as BDNF, MCP-1, IL-10, and TAC, which suggests that probiotics could synergistically act with other active ingredients of the supplement (virgin olive oil, prebiotic fiber, n-3 PUFA, or whey protein, etc) and with the Mediterranean dietary pattern to improve the proinflammatory and oxidative status, and FFM. Despite more studies being necessary to recommend the use of pre- and probiotics in patients with MHD, they could reduce the levels of solutes that contribute to the uremic syndrome, as well as improve the lipid profile, oxidative stress, and systemic inflammation (19, 20).

All these dietary changes in body composition and biomarkers were produced with no increase in the serum levels of phosphorus or potassium (with a clear tendency of the latter toward reduction and achieving normal values in the supplemented groups), in accordance with other randomized studies (1, 36). We also found vitamin D increases after 3 months, similar to the findings of other recent trials (5). These results suggest that this new ONS (with a low content of these electrolytes) can help to achieve nutritional requirements in these patients.

Interestingly, urea levels increased significantly in the SU-PL group but not in the SU-PR. Urea has recently been proposed as a relevant gut-derived toxin that triggers molecular changes leading to insulin resistance and endothelial dysfunction. In CKD models, probiotics from the genus Bifidobacterium have shown reductions in serum urea nitrogen and other uremic toxins levels (46). It is possible that in our study, the probiotics have contributed to the fact that urea levels do not rise despite the increase in protein intake with ONS.

The main limitation of the study (in part due to the SARS-CoV-2 pandemic and the associated difficulties in completing the patients’ follow-up) is that, although we did reach the planned sample size (more than 17 subjects per group), this may not be enough for some variables if we consider the high dropout rate. Nonetheless, the power for the variables reaching statistical significance was, in all cases, above 80%. Some statistical significances were only observed after adding the two supplemented groups (SU-TOT), which may be due to an additive effect of the SU-PR group but could also be secondary to the increase in n; the first option cannot be tested as there was no control-PR group. Notwithstanding, no patient withdrew from the trial because of gastrointestinal symptoms (which were similar to the C group during all the interventions), and only six because of lack of supplement acceptance (16% of the total number of patients randomized to ONS). These results are similar (or better) to those from other trials in which adherence was low (7, 47). Acceptance regarding the organoleptic characteristics of the supplement was high, which is in part motivated by the possibility of changing the flavor as, although the supplement is presented in vanilla flavor, it is delivered with six additional flavors that can be added to facilitate compliance, acceptance, and individualization. ONS compliance was self-reported, and no biomarker to evaluate intake was used; in this sense, it would have been better to measure the normalized protein catabolic rate (nPCR) to evaluate protein intake. Finally, no data on the acid–base status were collected, and it could have provided useful information.

As strengths of the study, we highlight the fact that it is a randomized clinical trial (double-blind regarding probiotics intake), the follow-up is in the long term (6 months), its multicentric nature, the measurement of multiple parameters (diet, morphofunctional nutritional assessment, biochemical parameters, and biomarkers of inflammation and oxidation), and the comparison with a C group that followed an individualized diet prescribed by registered dietitians.

## 5. Conclusion

The new ONS specifically designed for patients with MHD with malnutrition (or at risk) improved caloric-protein intake, nutritional status (especially FFM), and some biomarkers of inflammation and oxidation; the addition of probiotics could act synergistically with the ONS components to improve these biomarkers. This study sets the path for new randomized studies with a higher number of patients and, in the long term, confirms these preliminary results and assesses the efficacy of the new ONS in terms of morbidity.

## Data availability statement

The data presented in this study are available on reasonable request from the corresponding authors.

## Ethics statement

The studies involving human participants were reviewed and approved by the Research Ethics Committee provincial of Málaga. The patients/participants provided their written informed consent to participate in this study.

## Author contributions

GO contributed to the conceptual design of the research, funding acquisition, and drafted the manuscript. GO, FH, and MP contributed to the interpretation of the data. All authors contributed to the acquisition, analysis of the data, critically revised the manuscript, and agreed to be fully accountable for ensuring the integrity and accuracy of the study.

## Footnotes

1
https://adventiapharma.com/nutricion-clinica/aromas
