# Supplementary material for: Effect on nutritional status and biomarkers of inflammation and oxidation of an oral nutritional supplement (with or without probiotics) in malnourished hemodialysis patients. A multicenter randomized clinical trial “Renacare Trial”
Source: Front Nutr. 2023 Feb 3;10:1107869. doi: 10.3389/fnut.2023.1107869 (PMC9936863; doi:10.3389/fnut.2023.1107869)
Supplement: Supplementary file 1 [file Table_1.DOCX]

Supplementary Material

# Supplementary Tables

**Table S1:** Digestive Symptoms Scale

|  | **Control** | **SU-PL** | **SU-PR** | **SU-TOT** | **p-values** |
| --- | --- | --- | --- | --- | --- |
|  | **(n=11)** | **(n=10)** | **(n=10)** | **(n=20)** |  |
| (Basal) Nausea |  |  |  |  | 0.154 |
| mean ± ds | 1.8 ± 3.2 | 1.1 ± 1.7 | 0.1 ± 0.3 | 1 ± 2.1 |  |
| median (p25;p75) | 0 (0; 3) | 0 (0; 2) | 0 (0; 0) | 0 (0; 1) |  |
| (Basal) Vomiting |  |  |  |  | 0.114 |
| mean ± ds | 0 ± 0 | 0.6 ± 1.3 | 0 ± 0 | 0.2 ± 0.8 |  |
| median (p25;p75) | 0 (0; 0) | 0 (0; 0) | 0 (0; 0) | 0 (0; 0) |  |
| (Basal) Diarrhea |  |  |  |  | 0.961 |
| mean ± ds | 1.1 ± 2.8 | 0.9 ± 1.9 | 0.7 ± 1.6 | 0.9 ± 2.1 |  |
| median (p25;p75) | 0 (0; 0) | 0 (0; 0) | 0 (0; 1) | 0 (0; 0) |  |
| (Basal) Constipation |  |  |  |  | 0.426 |
| mean ± ds | 1.1 ± 1.7 | 1.4 ± 2 | 0.5 ± 1.2 | 1 ± 1.6 |  |
| median (p25;p75) | 0 (0; 2) | 0 (0; 3) | 0 (0; 0) | 0 (0; 2) |  |
| (minimum; maximum) | (0; 5) | (0; 5) | (0; 4) | (0; 5) |  |
| (Basal) Reflux |  |  |  |  | 0.804 |
| mean ± ds | 1 ± 3.2 | 0.8 ± 1.7 | 0.5 ± 1.8 | 0.8 ± 2.2 |  |
| median (p25;p75) | 0 (0; 0) | 0 (0; 0) | 0 (0; 0) | 0 (0; 0) |  |
| (Basal) Abdominal pain |  |  |  |  | 0.685 |
| mean ± ds | 1 ± 2.5 | 0.1 ± 0.3 | 0.5 ± 1.5 | 0.5 ± 1.7 |  |
| median (p25;p75) | 0 (0; 0) | 0 (0; 0) | 0 (0; 0) | 0 (0; 0) |  |
| (Basal) Bloating |  |  |  |  | 0.899 |
| mean ± ds | 0.9 ± 1.7 | 1.2 ± 2 | 1.2 ± 2.1 | 1.1 ± 1.9 |  |
| median (p25;p75) | 0 (0; 2) | 0 (0; 1) | 0 (0; 3) | 0 (0; 2) |  |
| (1) Nausea |  |  |  |  | 0.99 |
| mean ± ds | 0.2 ± 0.6 | 0.1 ± 0.3 | 0.1 ± 0.3 | 0.1 ± 0.4 |  |
| median (p25;p75) | 0 (0; 0) | 0 (0; 0) | 0 (0; 0) | 0 (0; 0) |  |
| (1) Vomiting |  |  |  |  | 1 |
| mean ± ds | 0 ± 0 | 0 ± 0 | 0 ± 0 | 0 ± 0 |  |
| median (p25;p75) | 0 (0; 0) | 0 (0; 0) | 0 (0; 0) | 0 (0; 0) |  |
| (1) Diarrhea |  |  |  |  | 0.683 |
| mean ± ds | 0.1 ± 0.3 | 0.5 ± 1.3 | 0.1 ± 0.3 | 0.2 ± 0.8 |  |
| median (p25;p75) | 0 (0; 0) | 0 (0; 0) | 0 (0; 0) | 0 (0; 0) |  |
| (1) Constipation |  |  |  |  | 0.278 |
| mean ± ds | 0.7 ± 1.6 | 0.8 ± 1.1 | 0.2 ± 0.6 | 0.5 ± 1.2 |  |
| median (p25;p75) | 0 (0; 0) | 0 (0; 2) | 0 (0; 0) | 0 (0; 0) |  |
| (minimum; maximum) | (0; 5) | (0; 3) | (0; 2) | (0; 5) |  |
| (1) Reflux |  |  |  |  | 0.997 |
| mean ± ds | 0.4 ± 1.3 | 0.1 ± 0.3 | 0.4 ± 1.2 | 0.3 ± 1 |  |
| median (p25;p75) | 0 (0; 0) | 0 (0; 0) | 0 (0; 0) | 0 (0; 0) |  |
| (1) Abdominal pain |  |  |  |  | 0.347 |
| mean ± ds | 0.6 ± 1.6 | 0 ± 0 | 0.5 ± 1.3 | 0.4 ± 1.2 |  |
| median (p25;p75) | 0 (0; 0) | 0 (0; 0) | 0 (0; 0) | 0 (0; 0) |  |
| (minimum; maximum) | (0; 5) | (0; 0) | (0; 4) | (0; 5) |  |
| (1) Bloating |  |  |  |  | 0.903 |
| mean ± ds | 0.8 ± 2.2 | 0.6 ± 1.3 | 0.9 ± 1.7 | 0.8 ± 1.7 |  |
| median (p25;p75) | 0 (0; 0) | 0 (0; 0) | 0 (0; 2) | 0 (0; 0) |  |
| (2) Nausea |  |  |  |  | 0.475 |
| mean ± ds | 0.3 ± 0.9 | 0.4 ± 0.7 | 0.4 ± 1.2 | 0.4 ± 1 |  |
| median (p25;p75) | 0 (0; 0) | 0 (0; 1) | 0 (0; 0) | 0 (0; 0) |  |
| (2) Vomiting |  |  |  |  | 0.35 |
| mean ± ds | 0 ± 0 | 0.1 ± 0.3 | 0 ± 0 | 0 ± 0.2 |  |
| median (p25;p75) | 0 (0; 0) | 0 (0; 0) | 0 (0; 0) | 0 (0; 0) |  |
| (2) Diarrhea |  |  |  |  | 0.378 |
| mean ± ds | 0.5 ± 1.6 | 0.7 ± 1.6 | 0.1 ± 0.3 | 0.4 ± 1.3 |  |
| median (p25;p75) | 0 (0; 0) | 0 (0; 1) | 0 (0; 0) | 0 (0; 0) |  |
| (2) Constipation |  |  |  |  | 0.758 |
| mean ± ds | 0.6 ± 1.3 | 0.5 ± 1.1 | 0.3 ± 0.9 | 0.5 ± 1.1 |  |
| median (p25;p75) | 0 (0; 0) | 0 (0; 0) | 0 (0; 0) | 0 (0; 0) |  |
| (2) Reflux |  |  |  |  | 0.555 |
| mean ± ds | 0.2 ± 0.4 | 0.4 ± 0.7 | 0.5 ± 1.5 | 0.4 ± 1 |  |
| median (p25;p75) | 0 (0; 0) | 0 (0; 1) | 0 (0; 0) | 0 (0; 0) |  |
| (2) Abdominal pain |  |  |  |  | 0.782 |
| mean ± ds | 0.5 ± 1.6 | 0.3 ± 0.7 | 0.2 ± 0.6 | 0.3 ± 1 |  |
| median (p25;p75) | 0 (0; 0) | 0 (0; 0) | 0 (0; 0) | 0 (0; 0) |  |
| (2) Bloating |  |  |  |  | 0.254 |
| mean ± ds | 0.2 ± 0.6 | 0.6 ± 0.8 | 1.2 ± 1.7 | 0.7 ± 1.2 |  |
| median (p25;p75) | 0 (0; 0) | 0 (0; 1) | 0 (0; 3) | 0 (0; 1) |  |
| (3) Nausea |  |  |  |  | 0.356 |
| mean ± ds | 0.7 ± 1.6 | 0 ± 0 | 0.6 ± 2.1 | 0.5 ± 1.5 |  |
| median (p25;p75) | 0 (0; 0) | 0 (0; 0) | 0 (0; 0) | 0 (0; 0) |  |
| (3) Vomiting |  |  |  |  | 0.403 |
| mean ± ds | 0 ± 0 | 0 ± 0 | 0.2 ± 0.6 | 0.1 ± 0.4 |  |
| median (p25;p75) | 0 (0; 0) | 0 (0; 0) | 0 (0; 0) | 0 (0; 0) |  |
| (3) Diarrhea |  |  |  |  | 0.196 |
| mean ± ds | 0.6 ± 1.3 | 1.6 ± 2.8 | 0.1 ± 0.3 | 0.7 ± 1.8 |  |
| median (p25;p75) | 0 (0; 0) | 0 (0; 2) | 0 (0; 0) | 0 (0; 0) |  |
| (3) Constipation |  |  |  |  | 0.756 |
| mean ± ds | 0.7 ± 1.6 | 1 ± 2 | 0.4 ± 0.8 | 0.7 ± 1.5 |  |
| median (p25;p75) | 0 (0; 0) | 0 (0; 1) | 0 (0; 0) | 0 (0; 0) |  |
| (3) Reflux |  |  |  |  | 0.843 |
| mean ± ds | 0.7 ± 1.6 | 0.9 ± 1.7 | 0.7 ± 1.6 | 0.8 ± 1.6 |  |
| median (p25;p75) | 0 (0; 0) | 0 (0; 2) | 0 (0; 0) | 0 (0; 0) |  |
| (minimum; maximum) | (0; 5) | (0; 5) | (0; 4) | (0; 5) |  |
| (3) Abdominal pain |  |  |  |  | 0.804 |
| mean ± ds | 0.6 ± 1.9 | 0.2 ± 0.4 | 0.2 ± 0.6 | 0.3 ± 1.1 |  |
| median (p25;p75) | 0 (0; 0) | 0 (0; 0) | 0 (0; 0) | 0 (0; 0) |  |
| (3) Bloating |  |  |  |  | 0.156 |
| mean ± ds | 0.6 ± 1.3 | 0.8 ± 1.6 | 2.5 ± 2.8 | 1.3 ± 2.2 |  |
| median (p25;p75) | 0 (0; 0) | 0 (0; 1) | 1 (0; 5) | 0 (0; 2) |  |
| (4) Nausea |  |  |  |  | 0.991 |
| mean ± ds | 0.4 ± 1 | 0.2 ± 0.4 | 0.5 ± 1.3 | 0.4 ± 0.9 |  |
| median (p25;p75) | 0 (0; 0) | 0 (0; 0) | 0 (0; 0) | 0 (0; 0) |  |
| (4) Vomiting |  |  |  |  | 0.368 |
| mean ± ds | 0 ± 0 | 0.1 ± 0.3 | 0 ± 0 | 0 ± 0.2 |  |
| median (p25;p75) | 0 (0; 0) | 0 (0; 0) | 0 (0; 0) | 0 (0; 0) |  |
| (4) Diarrhea |  |  |  |  | 0.152 |
| mean ± ds | 0.3 ± 0.9 | 0.8 ± 1.6 | 0 ± 0 | 0.4 ± 1.1 |  |
| median (p25;p75) | 0 (0; 0) | 0 (0; 1) | 0 (0; 0) | 0 (0; 0) |  |
| (4) Constipation |  |  |  |  | 0.528 |
| mean ± ds | 0.7 ± 1.6 | 0.8 ± 1.6 | 0.3 ± 0.9 | 0.6 ± 1.4 |  |
| median (p25;p75) | 0 (0; 1) | 0 (0; 1) | 0 (0; 0) | 0 (0; 0) |  |
| (4) Reflux |  |  |  |  | 0.839 |
| mean ± ds | 0.3 ± 0.9 | 0.3 ± 0.7 | 0.5 ± 1.1 | 0.4 ± 0.9 |  |
| median (p25;p75) | 0 (0; 0) | 0 (0; 0) | 0 (0; 0) | 0 (0; 0) |  |
| (4) Abdominal pain |  |  |  |  | 0.817 |
| mean ± ds | 0.4 ± 1.3 | 0.2 ± 0.6 | 0.3 ± 0.7 | 0.3 ± 0.9 |  |
| median (p25;p75) | 0 (0; 0) | 0 (0; 0) | 0 (0; 0) | 0 (0; 0) |  |
| (4) Bloating |  |  |  |  | 0.205 |
| mean ± ds | 0.3 ± 0.7 | 0.8 ± 1.3 | 1.7 ± 1.9 | 0.9 ± 1.5 |  |
| median (p25;p75) | 0 (0; 0) | 0 (0; 1) | 1 (0; 3) | 0 (0; 2) |  |
| (5) Nausea |  |  |  |  | 0.355 |
| mean ± ds | 0.3 ± 0.7 | 0.2 ± 0.6 | 0 ± 0 | 0.2 ± 0.5 |  |
| median (p25;p75) | 0 (0; 0) | 0 (0; 0) | 0 (0; 0) | 0 (0; 0) |  |
| (5) Vomiting |  |  |  |  | 1 |
| mean ± ds | 0.1 ± 0.3 | 0.1 ± 0.3 | 0.1 ± 0.3 | 0.1 ± 0.3 |  |
| median (p25;p75) | 0 (0; 0) | 0 (0; 0) | 0 (0; 0) | 0 (0; 0) |  |
| (minimum; maximum) | (0; 1) | (0; 1) | (0; 1) | (0; 1) |  |
| (5) Diarrhea |  |  |  |  | 0.996 |
| mean ± ds | 0.1 ± 0.3 | 0.3 ± 0.9 | 0.1 ± 0.3 | 0.2 ± 0.6 |  |
| median (p25;p75) | 0 (0; 0) | 0 (0; 0) | 0 (0; 0) | 0 (0; 0) |  |
| (5) Constipation |  |  |  |  | 0.18 |
| mean ± ds | 0.3 ± 0.7 | 1.1 ± 1.4 | 0.3 ± 0.7 | 0.6 ± 1 |  |
| median (p25;p75) | 0 (0; 0) | 0.5 (0; 2) | 0 (0; 0) | 0 (0; 1) |  |
| (5) Reflux |  |  |  |  | 0.996 |
| mean ± ds | 0.1 ± 0.3 | 0.1 ± 0.3 | 0.3 ± 0.9 | 0.2 ± 0.6 |  |
| median (p25;p75) | 0 (0; 0) | 0 (0; 0) | 0 (0; 0) | 0 (0; 0) |  |
| (5) Abdominal pain |  |  |  |  | 0.755 |
| mean ± ds | 0.3 ± 0.7 | 0.1 ± 0.3 | 0.2 ± 0.6 | 0.2 ± 0.6 |  |
| median (p25;p75) | 0 (0; 0) | 0 (0; 0) | 0 (0; 0) | 0 (0; 0) |  |
| (5) Bloating |  |  |  |  | 0.101 |
| mean ± ds | 0 ± 0 | 0.8 ± 1.6 | 1.1 ± 1.7 | 0.6 ± 1.4 |  |
| median (p25;p75) | 0 (0; 0) | 0 (0; 1) | 0 (0; 2) | 0 (0; 0) |  |
| (6) Nausea |  |  |  |  | 0.126 |
| mean ± ds | 0.3 ± 0.7 | 0 ± 0 | 0 ± 0 | 0.1 ± 0.4 |  |
| median (p25;p75) | 0 (0; 0) | 0 (0; 0) | 0 (0; 0) | 0 (0; 0) |  |
| (6) Vomiting |  |  |  |  | 0.126 |
| mean ± ds | 0.2 ± 0.4 | 0 ± 0 | 0 ± 0 | 0.1 ± 0.3 |  |
| median (p25;p75) | 0 (0; 0) | 0 (0; 0) | 0 (0; 0) | 0 (0; 0) |  |
| (6) Diarrhea |  |  |  |  | 0.671 |
| mean ± ds | 0.1 ± 0.3 | 0.7 ± 1.5 | 0.1 ± 0.3 | 0.3 ± 0.9 |  |
| median (p25;p75) | 0 (0; 0) | 0 (0; 0) | 0 (0; 0) | 0 (0; 0) |  |
| (6) Constipation |  |  |  |  | 0.168 |
| mean ± ds | 0.6 ± 1.1 | 0.8 ± 1.6 | 0 ± 0 | 0.5 ± 1.1 |  |
| median (p25;p75) | 0 (0; 1) | 0 (0; 1) | 0 (0; 0) | 0 (0; 0) |  |
| (6) Reflux |  |  |  |  | 0.213 |
| mean ± ds | 0.9 ± 1.7 | 0 ± 0 | 0.8 ± 1.8 | 0.6 ± 1.4 |  |
| median (p25;p75) | 0 (0; 2) | 0 (0; 0) | 0 (0; 0) | 0 (0; 0) |  |
| (6) Abdominal pain |  |  |  |  | 0.595 |
| mean ± ds | 0.1 ± 0.3 | 0 ± 0 | 0.2 ± 0.6 | 0.1 ± 0.4 |  |
| median (p25;p75) | 0 (0; 0) | 0 (0; 0) | 0 (0; 0) | 0 (0; 0) |  |
| (6) Bloating |  |  |  |  | 0.139 |
| mean ± ds | 0.5 ± 1.6 | 1.3 ± 2.1 | 1.7 ± 1.9 | 1.2 ± 1.9 |  |
| median (p25;p75) | 0 (0; 0) | 0 (0; 2) | 1 (0; 4) | 0 (0; 2) |  |

Data are presented as mean ± standard deviation (m± ds) and as median and interquartile range: med (p25; p75) maximum and minimum. SU-PL supplement group+ placebo; SU-PR supplement group+ probiotics; SU-TOT: supplement group (SU-PL+SU-PR). Score: scale from 0 (not at all) to 10 (very frequently).

**Table S2:** Supplement Acceptance

|  | **SU-PL** | **SU-PR** | **SU-TOT** |
| --- | --- | --- | --- |
|  | **(n=10)** | **(n=10)** | **(n=20)** |
| **3 months** | | | |
| Do you like the taste? m± ds | 7.3 ± 1.6 | 7.1± 1.4 | 7.2 ± 1.5 |
| Do you find it sweet? m± ds | 6.4 ± 2.3 | 6.5 ± 2.1 | 6.5 ± 2.2 |
| Do you find it salty? m± ds | 0.2 ± 0.6 | 0 ± 0 | 0.1 ± 0.4 |
| Do you find it bitter? m± ds | 0.6 ± 1.3 | 0 ± 0 | 0.29 ± 0.9 |
| Do you find it sour? m± ds | 0 ± 0 | 0.27 ± 0.9 | 0.14 ± 0.6 |
| After the intake, do you notice aftertaste? m± ds | 3 ± 3.43 | 2.82 ± 2.9 | 2.9 ± 3.11 |
| After the intake, do you feel full until the next meal? m± ds | 5.7 ± 3.1 | 8.09 ± 2.2 | 6.9 ± 2.9 |
| Do you like the way it smells? m± ds | 6 ± 3.5 | 6.1 ± 2.6 | 6.1 ± 3 |
| Do you like the look/color of the supplement? m± ds | 6.2 ± 1.9 | 6.4 ± 1.4 | 6.3 ± 1.6 |
| How do you feel after taking it? m± ds | 7.3 ± 1.7 | 7.4 ± 1.4 | 7.3 ± 1.6 |
| **6 months** | | | |
| Do you like the taste? m± ds | 6.9 ± 1.66 | 6.6 ± 1.35 | 6.7 ± 1.5 |
| Do you find it sweet? m± ds | 7 ± 2.4 | 7.1 ± 2.1 | 7.1 ± 2.2 |
| Do you find it salty? m± ds | 0.4 ± 1.3 | 0 ± 0 | 0.2 ± 0.9 |
| Do you find it bitter? m± ds | 0.3 ± 0.9 | 0 ± 0 | 0.15 ± 0.7 |
| Do you find it sour? m± ds | 0.6 ± 1.3 | 0 ± 0 | 0.3 ± 0.9 |
| After the intake, do you notice aftertaste? m± ds | 3 ± 2.8 | 2.5 ± 3 | 2.75 ± 2.8 |
| After the intake, do you feel full until the next meal? m± ds | 4.7 ± 2.4 | 7.5 ± 1.6 | 6.1 ± 2.5 |
| Do you like the way it smells? m± ds | 6.8 ± 2.2 | 6.1 ± 2.7 | 6.45 ± 2.4 |
| Do you like the look/color of the supplement? m± ds | 4.9 ± 1.4 | 5.8 ± 1.8 | 5.35 ± 1.7 |
| How do you feel after taking it? m± ds | 7.3 ± 1.7 | 6.8 ± 1.5 | 7.05 ± 1.6 |

Data are presented as mean ± standard deviation (m± ds). SU-PL supplement group+ placebo; SU-PR supplement group+ probiotics; SU-TOT: supplement group (SU-PL+SU-PR). No significant differences were observed between groups. Questions 1 to 9: scale from 0 (not at all) to 10 (a lot); question 10: 0 very badly to 10 very well 10.
